# Patient reported outcome measures for use in pregnancy and childbirth: a systematic review

**DOI:** 10.1186/s12884-019-2318-3

**Published:** 2019-05-06

**Authors:** Fiona Dickinson, Mary McCauley, Helen Smith, Nynke van den Broek

**Affiliations:** Centre for Maternal and Newborn Health, Liverpool School of Tropical Health, Pembroke Place, Liverpool, L3 5QA UK

**Keywords:** Patient reported outcome measure, Quality of care, Maternity care, Pregnancy, Childbirth

## Abstract

**Background:**

Globally, an increasing number of women give birth in a healthcare facility. Improvement in the quality of care is crucial if preventable maternal mortality and morbidity are to be reduced. A Patient Reported Outcome Measure (PROM) can be used to measure quality of care and provide new information on the impact that treatment or interventions have on patient’s self-assessed health and health-related quality of life. We conducted a systematic review to identify which condition-specific PROMs are currently available for use in pregnancy and childbirth, and to evaluate whether these could potentially be used to assess the quality of care provided for women using maternity services.

**Methods:**

We searched for articles relating to the use of PROMs related to care during pregnancy, childbirth, the postnatal period and women’s health more generally using PsycINFO, CINAHL, Medline and Web of Science databases as well as “grey literature”, with no date limit. Any PROM identified was reviewed with regards to development, use, and potential applicability to assess quality of maternity care provision. A narrative synthesis was used to summarise findings.

**Results:**

Six papers were identified; two related to aspects of pregnancy (hyperemesis gravidarum and gestational diabetes), and four related to childbirth and the postnatal period (obstetric haemorrhage and postnatal depression). Within these papers, a total of 14 different tools were identified, which assessed a variety of aspects of physical, psychological and social health, or were generic tools, not specific to childbirth. One PROM addressed childbirth generally, however, it did not ask for or provide specific outcome measures but required women to identify and then assess what they considered the most important areas in their life affected by childbirth.

**Conclusions:**

To date, there is no PROM agreed which would be suitable as patient reported outcome measure for the assessment of the quality of care women receive during pregnancy or after childbirth. However, there are a variety of available assessment tools which could potentially be helpful in developing new and existing PROMs for maternity care.

## Background

Improving quality of care and patient’s health outcomes are important goals for healthcare providers. In order to assess quality of care, women’s perspectives of care provision need to be further understood and taken into consideration [1]. There is also a need for better measurement of health outcomes as experienced by the individual and for this information to come from the individual patient or client themselves. The importance of the patient’s perspective and experience of care is increasingly recognised and takes into account efforts to improve the quality and effectiveness of health care. There is increasing support and recognition for the use of patient-reported outcome measures (PROMs), patient-reported experience measures (PREMs), and, assessment of patient satisfaction with care when assessing the quality of care with regard to clinical effectiveness, safety, and patient experience, and to guide service improvement [2, 3].

Currently, available PROMs are generally assessed via a series of structured questions that ask patients about specific symptoms, such as pain levels, or aspects of their health or health related quality of life. They allow the person experiencing the health outcome to gauge its severity. It has been shown that physicians may under-estimate symptom severity and the impact of treatments or care interventions on patients [4]. The use of one or more PROMs may help to address this. Questionnaires or tools for the assessment of PROMs do not ask about patients’ satisfaction with, or experience of, healthcare services, or seek their opinions about how successful their treatment was but ask about specific outcomes of the care or intervention received. The data they generate can be used for a number of purposes including to guide clinical decision making, promote patient choice, direct the allocation of resources, standardise research outcomes, and, assess the quality of care [2].

PREM gather information on patients’ views of their experience whilst receiving care. They can be an indicator of the quality of patient care received. In contrast to PROMs however, PREMs do not look at the outcomes of care but the impact of the process of the care on the patient’s experience e.g. communication and timeliness of receiving care. They differ from satisfaction surveys by reporting objective patient experiences. In general, PREMs measure the process of care provision, while PROMs are measures of clinical care effectiveness [5].

Globally, the number of women who deliver in a healthcare facility or with a skilled birth attendant has increased to 72% overall [6]. With improved availability and coverage of maternity care worldwide, further reductions in mortality and morbidity associated with pregnancy and childbirth will require an increase in quality of maternity care services [6, 7]. Improvements in the quality of maternity care have the potential to improve maternal and newborn health outcomes directly through better quality, more effective care, and, indirectly through improved perception and experience of care, and, associated increased uptake of maternity services.

Efforts to improve the quality of health care are also essential, especially in low resource settings, to ensure limited resources can be used most effectively. PROMs related to maternity care have the potential to provide a measure or benchmark of care outcomes and could potentially be used to monitor quality over time or across different settings and different levels of a health system.

The aim of this systematic literature review was to identify if there are existing PROMs relating to health outcomes experienced by women during and after pregnancy or childbirth. We sought to identify any existing maternity PROMs currently available; and to subsequently assess if these might be suitable for evaluating the quality of care and/or contribute to the development of new maternity PROMs for this purpose.

## Methods

### Search strategy

A PROM for the purposes of this review was characterised as 1) patient reported (either by self-completion of an assessment tool or, where necessary, patients are asked the questions by a third party), and, 2) assessing health, or, health-related quality of life. To optimize the search strategy and identify appropriate and relevant search terms, an initial search was carried out using “Patient Reported Outcome” with various terms relating to “pregnancy” and “childbirth”. This highlighted alternative search terms such as: “patient recorded outcome”, “patient reported outcome” and “patient related outcome”.

**Fig. 1:**
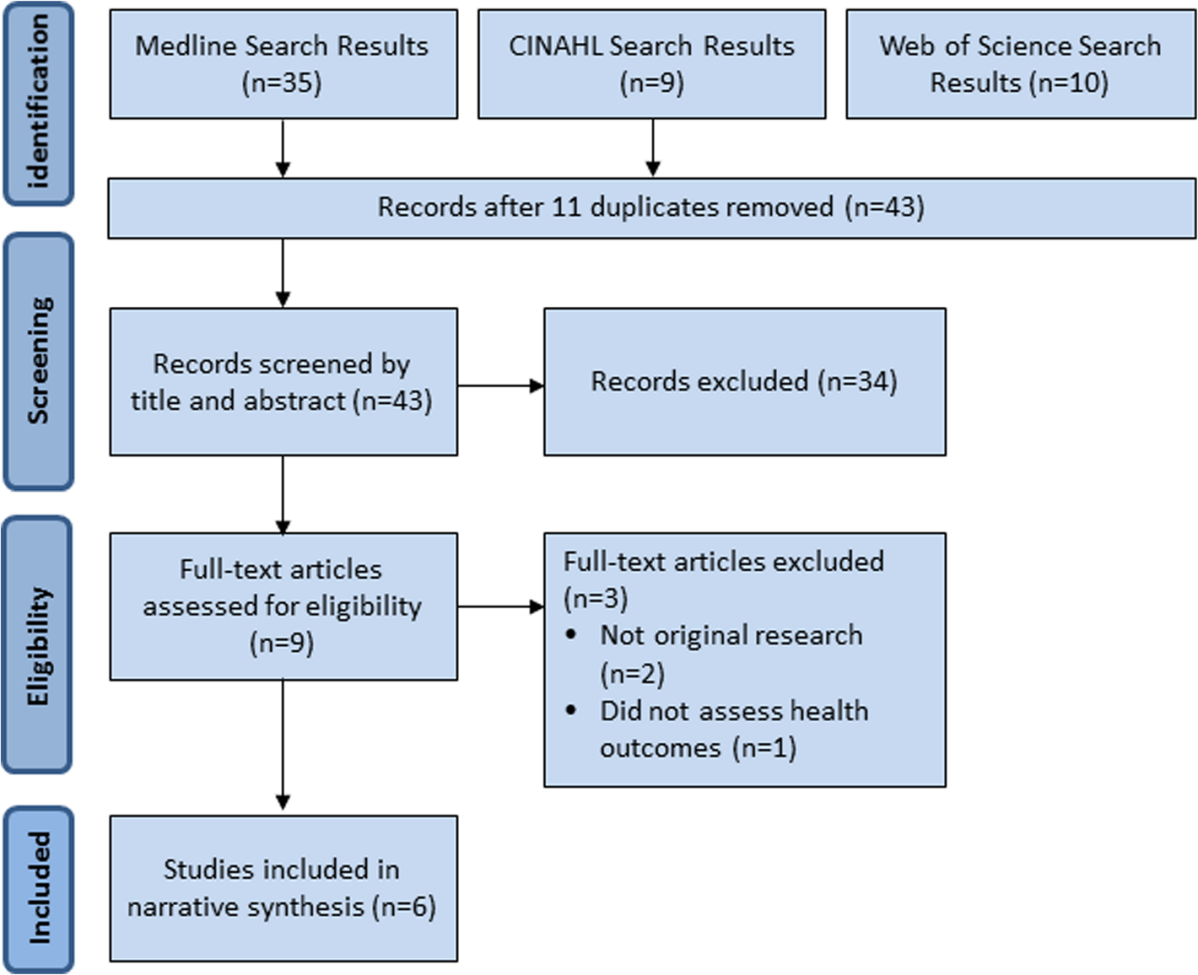
PRISMA flow diagram

Consequently, the developed search strategy was used to search Medline (1949–2018), CINAHL (1937–2018), PsycINFO (1887–2018) and Web of Science (1898–2018) using the following search terms: Patient report* outcome* OR Patient recorded outcome* OR patient related outcome* AND Matern* OR Pregnan* OR Natal OR Birth/parturition OR Obstet* OR Women’s health. Results were limited by English language and included MESH headings as applicable. Asterisks were used where appropriate to allow for alternative word endings such as plurals and maternal/maternity. No time limitation was applied to the search and it was most recently carried out in May 2018. Filters were used to exclude non-English language studies and those relating to children or men. A search of the grey literature was also carried out (Fig. 1).

As the review sought to identify any PROMs used in studies rather than study outcomes per se, frameworks such as PICO (Population, Intervention, Comparison, Outcome) (Methley et al. 2014) were of limited applicability. However, conceptually, the first two aspects were used, where the population included women during pregnancy or childbirth and the intervention was the application of a Patient Reported/Recorded Outcome Measure.

In addition, a search was carried out of the CROWN Initiative (Core Outcomes in Women’s and Newborn Health), COMET (Core Outcome Measures in Effectiveness Trials), and PROMIS (Patient-Reported Outcomes Measurement Information System) websites, to identify any existing PROMs that would meet the inclusion/exclusion criteria.

### Study selection

All citations identified were independently screened by two researchers, by title and abstract with discrepancies resolved by a third researcher. Full text versions of all potentially included studies were obtained and reviewed, and the results compared and mutually agreed.

Inclusion criteria were; primary research studies detailing use or development of a PROM relating to pregnancy, childbirth or ‘women’s health’. Studies were excluded if they described using patient reported experience or satisfaction measures only or if they did not directly address or were related to pregnancy or childbirth.

### Quality assessment and synthesis

Included studies were analysed and presented using textual narrative synthesis. Many frameworks for assessing quality of included studies in systematic reviews focus on assessing the risk of bias when conducting the studies [NHLBI, NOS, QUADAS-2] [8–10], including patient selection, sample size, and blinding of researchers. As the primary focus of this review was to identify PROMs used rather than comparing study outcomes, these were not appropriate. The quality of studies was thus assessed pragmatically with regard to the clarity and consistency of concepts [11].

Note was taken of any comments relating to the quality and usability of the PROMs employed. Any identified PROMs were assessed regarding the degree to which they could be used in assessing maternity care, and/or individual aspects of childbirth.

Included studies were summarised in a predesigned summary table (Table 1). Data obtained included; study, setting, population type and study design, and aspects of health addressed, as well as the specific tools used to measure the outcomes. Studies were further assessed to determine whether outcomes measured might be useful in assessing quality of care. The specific outcomes measured by the tools and the format including number and type of questions were also extracted (Table 2).

**Table 1:** Summary table of included studies of Patient Reported Outcomes (PROM)

Reference	Country	Population and study design	Aspect of pregnancy and childbirth assessed	Setting or level of care	Aim of study	Tools used in study to assess health outcomes
Fletcher et al. (2015) [15]	UK	273 women admitted to hospital with hyperemesis gravidarum were randomised to receive either individualised or usual care and advice.	Pregnancy - Antenatal care	Hospital in-patient	Using HIS to tailor advice on hyperemesis to individual women’s needs and reduce hospital admissions.	• Hyperemesis Impact of Symptoms (HIS) • SF-36 • EQ-5D-3 L • Pregnancy Unique Quantification of Emesis (PUQE)
Kopec et al. (2015) [12]	Poland	205 women with gestational diabetes treated at a clinic in Poland were assessed twice during pregnancy with an average eight-week interval.	Pregnancy - Diabetes in pregnancy	Clinic	Investigate changes in PROs (particularly psychological and social) of women with GDM during pregnancy and identify factors associated with distress.	• SF-8 (short version of the SF-36) • Hospital Anxiety and Depression Scales (HADS) • Problem Areas in Diabetes (PAID)
Symon et al. (2015) [17]	UK	Tool was posted to 678 women recruited to a RCT of self-hypnosis for pain during birth to assess as part of a 10-page pack.	Childbirth – not specified	Home	Assess the feasibility and acceptability of using the Mother Generated Index and compare its findings with other QoL tools.	• Mother Generated Index (MGI) • EQ-5D-3 L • Edinburgh Postnatal Depression Scale (EPDS) • State Trait Anxiety Inventory (STAI)
Thompson et al. (2011) [13]	Australia & New Zealand	A multi-centre cohort study including 206 women with significant primary postpartum haemorrhage (>1500mls).	Postnatal – Postpartum haemorrhage	Home	To describe the physical and psychological outcomes of women who had experienced a significant primary postpartum haemorrhage.	• Edinburgh Postnatal Depression Scale (EPDS) • State-Trait Anxiety Inventory (STAI) • PTSD checklist • Milligan’s postpartum fatigue scale • SF-36
Visser et al. (2018) [14]	Netherlands	A retrospective, cross-sectional survey of 372 women who had experienced major obstetric haemorrhage (>2500mls) in six hospitals.	Maternity –Major obstetric haemorrhage	Home	To explore patients’ experience and outcomes following major obstetric haemorrhage and to investigate which patients are most at risk of negative sequelae.	• Study specific (based on Consumer Assessment of Healthcare Providers and Systems)
Yawn et al. (2012) [16]	USA	2343 women between 5 and 12 weeks postnatal, whose general practice was randomly allocated to provide either ‘usual care’ or the intervention package.	Maternity – Postnatal depression	Primary care	Determine the effect of a primary care based screening, diagnosis and management intervention on postnatal depression in women 5–12 weeks postpartum.	• Edinburgh Postnatal Depression Scale (EPDS) • 9-item Patient Health Questionnaire (PHQ-9)

Abbreviations: *EPDS* = Edinburgh Postnatal Depression Scale; *HADS* = Hospital Anxiety and Depression Scales; *HIS* = Hyperemesis Impact of Symptoms; *MGI* = Mother Generated Index; *PAID* = Problem Areas in Diabetes; *PHQ*-9 = 9-item Patient Health Questionnaire; *PUQE* = Pregnancy Unique Quantification of Emesis; *SF*-36 = Short Form 36; *STAI* = State-Trait Anxiety Inventory

**Table 2:** Data collection tools used in included studies and health outcomes measured

Tool	Number of questions	Areas covered by tool	Topics covered
Hyperemesis Impact of Symptoms (HIS) [15]	10	Physical & psychological	Nausea, vomiting, tiredness, emotional state, anxiety
Pregnancy Unique Quantification of Emesis and Nausea (PUQE) [15]	3	Physical	Nausea, vomiting, retching
EQ-5D-3 L [15, 17]	5 + 1	Generic	
SF36 [13, 15]	36	Generic	
Hospital Anxiety & Depression Scale (HADS) [12]	14	Psychological	Anxiety, depression
Problem Areas in Diabetes (PAID) [8]	20	Psychological	Anxiety, depression, loneliness, anger
SF8 [12]	8	Generic	
Study specific [12]		Physical and social	Pain, fatigue, diet, exercise, insulin injection frequency
Mother Generated Index (MGI) [7]		Open	N/A
Edinburgh Postnatal Depression Scale (EPDS) [13, 16, 17]	10	Psychological	Postnatal depression
State Trait Anxiety Inventory (STAI) [13, 17]		Psychological	Anxiety
Milligan’s postpartum fatigue scale [13]		Physical & psychological	Fatigue
PTSD checklist [13]		Physical & psychological	Post-traumatic stress disorder
9-item Patient Health Questionnaire (PHQ-9) [16]	10	Psychological	Severe depression
Study specific [14]	44	Physical & psychological	

## Results

No studies were included from CROWN or COMET initiatives, as these were found to focus on core outcome sets for reporting clinical study data rather than on patient reported outcomes. The searches produced a total of 54 papers (Fig. 1). Following screening by title, abstract and full text, a total of six studies were included. The studies addressed various aspects of pregnancy and childbirth including gestational diabetes, hyperemesis gravidarum, postpartum haemorrhage and postnatal depression (Table 1). The PROMs were used in three main ways: to assess the impact of disease or health complication (postpartum haemorrhage) [12–14]; to assess the impact of an intervention such as a specific care package [15, 16]; or to assess the acceptability of a new PROM [17].

Most of the tools addressed either physical symptoms (e.g. pain, vaginal bleeding, or signs of infection) or psychological aspects (e.g. depression, anxiety, loneliness) of women’s health or a combination of both. One study however, also included outcomes relating to the social aspects of women’s quality of life such as the impact that gestational diabetes could have on women’s personal, social and work life [12].

### PROMs during pregnancy

Of the six included studies, two looked at specific medical conditions relating to pregnancy: hyperemesis (Fletcher et al) and gestational diabetes (Kopec et al) [12, 15]. Fletcher’s study assessed interventions to reduce the impact of hyperemesis gravidarum by means of a randomised controlled trial [15]. The intervention consisted of assessing the effect of hyperemesis on pregnant women using the previously validated Hyperemesis Impact of Symptoms (HIS) tool, and with this information providing advice tailored specifically to each woman’s needs. Women’s ability to cope with their symptoms were then assessed using a variety of methods, including the Pregnancy Unique Quantification of Emesis (PUQE), a subscale of the SF-36, and the EQ-5D (Table 2), alongside rates of hospital readmissions, over a six-week period.

In order to determine the changes women with gestational diabetes experienced during their pregnancies, and to identify the factors associated with distress in women, Kopec et al. used a range of PROMs including the condition-specific Problem Areas in Diabetes (PAID), Hospital Anxiety and Depression Scales (HADS) and the generic Short Form 8 (SF8) [12].

Both of these studies addressed conditions commonly experienced during pregnancy using a combination of condition specific and generic PROMs, and cover aspects of physical and psychological health.

### PROMs following childbirth

Four of the included studies explored the effects of childbirth on the women, during the postnatal period, focussing largely on postpartum haemorrhage and postnatal depression. Both Thompson et al. and Visser et al. explored the impact of severe postpartum haemorrhage following childbirth [13, 14]. In a similar way to the two pregnancy related papers, Thompson et al. in their prospective study, used a variety of widely recognised, condition specific and generic PROMs (Table 2) to explore the effect of primary postpartum haemorrhage (PPH) (defined in their study as greater than 1500 ml) on women, over a four-month period following childbirth. Conversely, Visser et al. developed a questionnaire for their retrospective study, based on the Consumer Assessment of Healthcare Providers and Systems (CAHPS, an American developed PREM), the Consumer Quality Index (a Dutch translation of the CAHPS), and previous interviews with 11 women who had experienced major obstetric haemorrhage (defined by the authors as > 1500 ml) [14]. The tool combined patient reported experience and outcomes and was administered to women between approximately 6 months and 6 years following the haemorrhage.

The study by Yawn et al. assessed the effect of an intervention providing additional education and diagnostic tools to primary care sites, on rates of diagnosis, and uptake, of therapy for, postpartum depression [16]. In this study a multi-step screening and diagnosis process used the Edinburgh Postnatal Depression Scale (EPDS) as an initial screening tool, followed by the administration of the Patient Health Questionnaire-9 (PHQ-9) and physician evaluation for those scoring > 10 on the EPDS. The EPDS is a tool developed in the 1980’s specifically for screening women for possible symptoms of depression during and after pregnancy. However, the PHQ-9 was not specifically designed for use in pregnancy/postpartum and it was felt by the authors to be more specific for assessing major depressive disorders. This study solely focussed on the psychological outcomes of childbirth using well recognised, condition specific tools.

Similar to some of the other studies, Symon et al. [17],, used several different tools (including generic and condition-specific), but was the only study to explore maternity care in general rather than a specific pregnancy or childbirth related condition. The primary purpose of the study was to investigate the feasibility and acceptability of using the recently developed Mother Generated Index (MGI) [17] as part of a randomised controlled trial (RCT) looking at the use of self-hypnosis for intrapartum pain. The authors compared the MGI with other widely used tools (EQ-5D-3 L, EPDS, Satisfaction with Life Scale, and State Trait Anxiety Index). The MGI was adapted from a previous tool – the Patient-Generated Index – to make it relevant to the context of maternity care. A key feature of the MGI was that the outcomes within the questionnaire were not pre-specified but were self-selected and generated by the person completing the assessment tool. A few suggestions were provided including: social life, work, weight gain, physical problems like backache. Completion of the questionnaire followed a three-step process, where step one allowed the woman to record the five most important areas of her life affected by the birth of her child. In step two the woman scored each area mentioned in step one in terms of how much (or not) she had been affected by it over the last month. Scores for each area could range from 10 indicating it is “Exactly as you would like to be” to 0 (“The worst you could imagine”). When totalled, these produced a quality of life index which could be used for comparison purposes across groups of women. Finally, in step three the woman allocated 12 ‘spending points’ indicating which of the areas cited were the most important and she would most like to see ‘improved’.

### Assessing the quality of maternity care

Of the six studies assessing pregnancy and childbirth, Fletcher et al. [15], Kopec et al. [12], Thompson et al. [13], and Yawn et al. [16], all addressed specific aspects of health in pregnancy or the postpartum period. As such, whilst the condition-specific questionnaires they used might be useful in assessing the quality of specialist care provided to individual patients, they were unlikely to be appropriate in addressing the quality of maternity care in women who do not experience these complications.

The MGI used by Symon et al. was the only tool used to assess maternity care more broadly [17]. However, its focus was oriented to assessing Quality of Life outcomes as part of a RCT and the specific areas assessed by the tool were left to the discretion of the women completing it. Therefore, women might decide to include issues not related to their health or the impact that the health care they received had on their quality of life. This being the case, it was thought unlikely to be of use in assessing the quality of maternity care provided at a health service level.

### Contributions to a new maternity PROM

None of the PROMs used in the identified studies were felt to be suitable for measuring the overall quality of care provided during pregnancy or after childbirth, in women who did not have the specific complications addressed. However, it was felt that a number of the tools used in the included studies might be of use in contributing to the development of new Maternity PROM (Table 2).

## Discussion

### Statement of principal findings

The studies included in this systematic review assessed a number of aspects of pregnancy and childbirth using a range of different condition-specific and generic PROMs. However, five of the six identified studies and the PROMs they deployed only addressed a single aspect of pregnancy or the postnatal period. The only PROMs to address childbirth more broadly did not specify the outcomes to be assessed and was therefore felt to be less useful in assessing quality of care at a health system level.

Apart from one study (Yawn et al) [16], all the studies included in this review assessed at least two of three areas of women’s health: physical, psychological and social. This reflects the need for any effective assessment tool to address more than just physical symptoms. This is likely to be particularly relevant to women during and after pregnancy when there is a significant impact of pregnancy and birth on a woman’s emotional, physical, psychological and social well-being.

### Strengths and weaknesses of the study

This review appears to be the first published review of PROMs used in pregnancy and childbirth. As a means of assessing the quality of care provided to women and thus contribute to its improvement, PROMs can potentially have a valuable role in enhancing care provision, particularly for women most in need, in low and middle-income countries. With the increases in hospital-based childbirth in these countries, there is a great need to ensure that the care provided by hospital staff is such that it meets the health needs of the women and is of such a standard that women will be further encouraged to attend health facilities to give birth rather than seeing it as just a slightly safer option than delivering at home with a traditional birth attendant.

A further consideration of the PROMs identified in this review is that they were all developed in a few high-income countries (USA, UK, Netherlands, Poland and Australia & New Zealand). This may limit their applicability in other settings, particularly in low-resource settings, and highlights the need for future PROMs to be developed and validated for use in LMICs as well.

PROMs are a relatively recent phenomenon, with little if anything published before the year 2000. Thus, one of the challenges of this review was the small number of studies available and this novelty also seems to be reflected in some ambiguity as to what constituted a PROM. The initial search did identify some questionnaires largely comprised of questions relating to patient satisfaction that had been described as PROMs. This made the primary search and abstract review a challenge, to identify which studies actually contained a tool which met our definition of a PROM. Another potential weakness of this review was the restriction of the search to papers published in the English language.

### Relationship of main findings to other studies

Some of the papers identified contained data collection tools which included patient satisfaction and patient experience questions as well as patient outcomes. For example, the study specific questionnaire used by Visser et al. also asked questions about information supplied and attention from staff [14]. This seemed to be an issue, not just in some of the studies included in this review but in the wider PROM literature.

This is the first paper to systematically explore what PROMs available and if they could be used for pregnancy and childbirth. Whilst the use of PROMs in assessing quality of care has been previously discussed and documented [2], there was no evidence of this being the case for pregnancy and childbirth. There is currently a need for the development of PROMs suitable for this purpose, to aid the assessment of quality of care in maternity service provision, and, to guide the work of clinicians and policy makers.

### Implications for clinicians, policymakers and future research

The findings of this review have research implications and contribute to the ongoing debate on the need for PROMs to assess quality of care for women during and after pregnancy and childbirth. To date, a range of methods and tools have been used to assess women’s health in general, each with their strengths and weaknesses. Issues such as cultural applicability, language and literacy need to be addressed when developing and deploying PROMs in non-Westernised and low-income settings.

The review highlights how different types of PROMs could be developed and used to capture women’s reported health outcomes during pregnancy and following childbirth. There is a need for a validated data collection tool that allows for consistent, standardised measurement of patient outcomes related specifically to women, during this important and complex life event.

## Conclusion

At present, there is no single PROM or composite of outcome assessments that has been developed for the purpose of assessing care quality provided to women during pregnancy and/or childbirth. It is important that the development of one or more outcome measures is based on existing evidence of development and use of PROMs in other areas of care. The development of a relatively simple and short suite of PROMs to assess patient reported outcomes for pregnancy and childbirth, is recommended.

## Abbreviations

COMET: Core Outcome Measures in Effectiveness Trials
CROWN: Core Outcomes in Women’s and Newborn Health
PPH: Post Partum Haemorrhage
PREM: Patient Reported Experience Measure
PROM: Patient Reported Outcome Measure
PROMIS: Patient-Reported Outcomes Measurement Information System
RCT: Randomised Controlled Trial
